# Bio-Based and Sustainable Alternatives to Conventional and Synthetic Leather

**DOI:** 10.3390/ma19061198

**Published:** 2026-03-18

**Authors:** Ewa Oleksińska-Merida, Michał Puchalski, Lucyna Herczyńska

**Affiliations:** Textiles Institute, Faculty of Textiles and Design, Lodz University of Technology, Żeromskiego 116, 90-924 Lodz, Poland; ewa.oleksinska-merida@dokt.p.lodz.pl (E.O.-M.); michal.puchalski@p.lodz.pl (M.P.)

**Keywords:** biomaterials, eco-leather, mycelium, biodegradable, sustainable, eco-friendly textiles

## Abstract

Growing demand for sustainable materials has intensified research into eco-friendly alternatives to conventional and synthetic leathers. Traditional bovine leather and its chromium-tanning process heavily contribute to water pollution, toxic waste generation, and carbon emissions, while synthetic leather derived from Polyvinyl Chloride (PVC) and polyurethane (PU) presents challenges related to fossil fuel dependence and non-biodegradability. This review explores bio-based and sustainable leather substitutes that are made of plants, microbial cellulose, and mycelium fungi. Plant-based leather substitutes such as Vegea^®^, Desserto^®^, and Piñatex^®^ use agricultural waste products to create durable, partially biodegradable composites. Microbial cellulose from kombucha fermentation offers material with good physical and aesthetic properties. Mycelium leather, derived from fungal biomass, demonstrates potential for scalable and low-impact production. Comparative analyses of mechanical and physical properties show that mycelium composites are approaching industrial standards, though challenges remain regarding tensile strength, water resistance, and process standardization. Despite current limitations, bio-based leathers, particularly mycelium composites, offer a promising way toward circular material innovation and carbon-neutral manufacturing in fashion, automotive, design and other industries.

## 1. Introduction

Sustainable materials represent a very important component in a circular economy and in meeting international climate commitments such as the Paris Agreement’s goal of net-zero emissions by 2050. They are produced without depleting non-renewable resources or causing ecological disruption. These materials can be bio-based (polymers, carbons, ceramics, etc.) or recycled, i.e., can be reprocessed or reused without requiring additional mining and mineral depletion. Scientifically, their sustainability depends on three important criteria: they must have functional durability and cost-efficiency across their lifecycle; be manufactured with minimal inputs of energy, water, and toxic elements; and offer viable pathways for reuse, recycling, or biodegradation at end-of-life. Research and innovation in this field are essential to reduce the environmental footprint of industrial processes while supporting scalable solutions for a low-carbon future [1].

The textile industry has an important role in modern life, with fabrics shaping numerous aspects of modern life, from the clothes we wear to the furniture we use, medical supplies, protective gear, and even elements of construction and transportation. However, this industry carries a significant environmental burden that continues to grow.

Textile consumption across the European Union (EU) has become one of the largest contributors to environmental degradation and climate change. The industry is particularly problematic when it comes to water and land use—textile production requires vast amounts of fresh water for dyeing and processing, while cultivation of raw materials like cotton takes up enormous areas of farmland, often leading to deforestation and soil depletion. Additionally, textiles are one of the highest contributors to the consumption of primary raw materials, emitting substantial greenhouse gases throughout their lifecycle [2,3].

One of the most alarming aspects of textile waste is the volume of discarded clothing. Each year, around five million tons of clothing are thrown away within the EU—equivalent to approximately 12 kg per person. Despite increasing awareness of the environmental consequences, only about 1% is recycled back into new clothing. The rest mostly ends up in landfills or incinerators, contributing to pollution and further climate change. A major challenge is that many clothes are made from synthetic fibers, which do not naturally decompose and can take hundreds of years to break down [4].

The environmental degradation attributed to the global textile industry is in many ways intensified by the production of animal leather. While the EU struggles with five million tons of annual textile waste, the leather sector introduces a set of complications that make the transition to a circular model more difficult. Although leather is frequently marketed as a natural byproduct, its conversion from a biological hide into a chemically stable industrial material involves high-intensity processing that often exceeds environmental degradation associated with cotton and polyester. This transformation requires a resource-heavy pipeline of water and land use, positioning leather as a significant contributor to the textile industry’s overall ecological footprint. Consequently, the leather industry functions as a critical subset of the textile crisis—sharing the same challenges of overproduction and waste but adding the specific burdens of livestock-driven deforestation and heavy-metal toxicity. Addressing the goals of the Paris Agreement, therefore, requires not only a reduction in synthetic waste but also a fundamental reimagining of high-impact materials like leather through the development of sustainable, bio-based alternatives.

## 2. Conventional and Synthetic Leather Markets

Traditional bovine leather, which is widely used in fashion, furniture, and the automotive industry, also has significant environmental consequences. Animal leather production heavily relies on animal slaughter, excessive water usage, and chemical treatments, generating toxic wastewater and soil contamination.

Currently, over 85–90% of global leather production is based on chrome tanning, the most common form of mineral tanning [5,6]. Processing one metric ton of raw hides generates approximately 600 kg of solid waste and 15–50 m^3^ of wastewater, containing around 250 kg of chemical oxygen demand (COD) and 100 kg of biochemical oxygen demand (BOD) [7]. Additionally, approximately 500 kg of chemical agents are introduced during processing. Because most tanning operations are water-based, wastewater management remains one of the most critical environmental challenges in tanneries [8].

Although cleaner tanning technologies are under development, such as the use of aluminum syntans in combination with chromium salts [9], the use of vegetable tanning agents [10], or tanning without using clean water [11], the environmental impact of tanning remains significant. This is because chrome tanning is preferred by most tanners, as chrome-tanned leathers are characterized by top quality, high hydrothermal stability and very good user properties in addition to the shorter time required to produce finished leather. Chrome tanning is also more economical than vegetable leather tanning [12].

**Figure 1:**
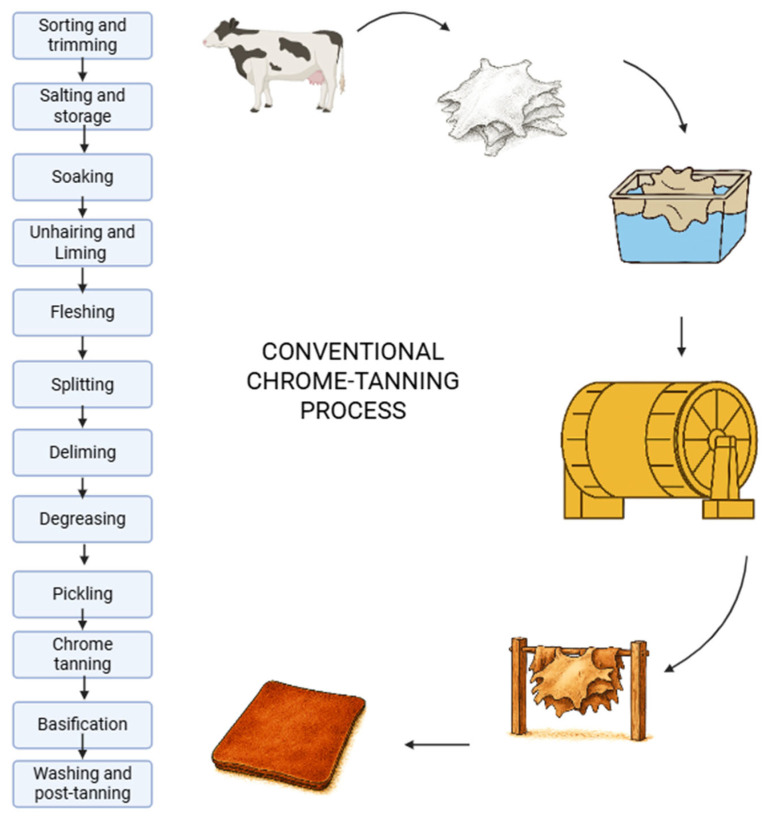
Schematic of conventional chrome-tanning process.

The conventional chrome-tanning process can be divided into several steps, as demonstrated in Figure 1. In the first step of the process, hides are sorted by size, weight, quality, and animal gender at various stages of the supply chain. Non-conforming hides may be redirected to alternative processing streams. Next, non-essential anatomical parts (e.g., limbs, tails, heads) are removed, generating regulated animal by-products, and this part of the process is called trimming. Afterwards hides are salted and stored. Salting stabilizes hides before further processing. Long-term methods, which include dry salting, brine soaking, drying, or brine drying (up to 6 months), are typically used for international trade. Whereas short-term methods such as cooling with ice, refrigerating, or biocidal treatment (2–5 days) are used for local supply chains [7].

The next step is soaking, which rehydrates hides and removes contaminants. It is conducted in two phases: pre-soaking and main soaking. Additives such as biocides, surfactants, and enzymes may be used to enhance cleaning and prevent microbial growth. Afterwards, hair, epidermis, and proteins are removed using sodium sulfide (Na_2_S or NaHS) and lime in a process called unhairing and liming (for bovine hides). In sheep hides, a sulfide–lime paste is applied to the flesh side to degrade hair follicles and facilitate wool removal. After wool extraction, hides undergo liming similar to bovine hides. In the next step, called fleshing, residual tissue and fat are mechanically removed using rollers and spiral blades. This process generates wastewater rich in organic matter [7].

The following step, called splitting, standardizes hide thickness via horizontal separation along the grain or flesh layer. It is performed in either a limed or tanned state using band splitters. Subsequent deliming removes residual alkalis by gradually lowering pH using water, weak acids, or salts (e.g., ammonium chloride, boric acid). Full deliming yields softer leather; partial deliming results in firmer material. Degreasing eliminates residual hair bulbs and pigments using commercial proteolytic enzymes, particularly in fine leather production [7].

Pickling starts the main tanning process, where hides are immersed in a solution of sodium chloride and sulfuric acid to lower pH to 2.8–3.0. This prepares collagen fibers for chrome penetration and prevents acid swelling. Basic chromium sulfate is introduced into the pickled hides. At low pH, chromium complexes penetrate the collagen matrix. The process is typically conducted in rotating drums to ensure uniform distribution. Basification (fixation) takes place when pH is gradually increased (using sodium bicarbonate or magnesium oxide) to 3.8–4.0, causing chromium to precipitate and bind to collagen fibers [10,12].

Washing and post-tanning operations include the removal of excess chemicals. Then leather undergoes neutralization, dyeing, fat liquoring, and finishing [10,12].

## 3. Synthetic-Leather-Coated Textile

Synthetic leather substitutes are produced from textiles coated with polyvinyl chloride (PVC) or polyurethane (PU). They have found a wide market because they reduce social and environmental concerns typically associated with leather production. However, they also require the use of hazardous chemicals in their production and are derived from fossil fuels, resulting in a lack of biodegradability, and have the same limited end-of-life options as most plastics [13,14].

**Figure 2:**
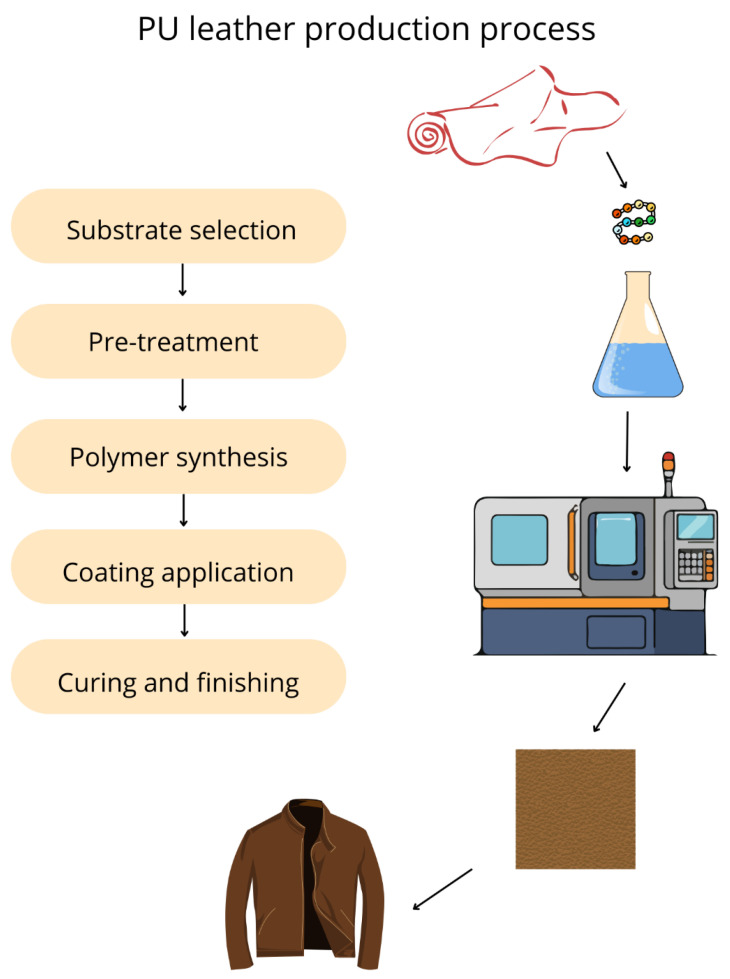
Schematic of PU leather production process.

These materials are typically manufactured by coating or laminating polymer layers onto a fabric base, commonly polyester or cotton, using techniques such as knife coating, casting, calendering, or the release paper method (Figure 2). In the latter, PU or PVC layers are applied to a casting paper, followed by foaming and bonding steps to produce a composite material with improved tactile properties and durability [15,16]. PU leather is generally softer, more breathable, and considered less environmentally damaging than PVC, as it contains fewer plasticizers and stabilizers. PVC leather has greater environmental risks due to the potential release of toxic compounds such as dioxins and chlorinated by-products during manufacturing and disposal.

Therefore, there is an urgent demand for sustainable and eco-friendly alternatives. In recent years, scientists and companies have been working on developing sustainable alternatives to animal-derived and synthetic leather. The production techniques for developing those bio-leathers vary depending on the source of cellulose utilized due to the confidentiality surrounding the businesses.

## 4. Current Trends in Alternative Bio-Leather Materials

### 4.1. Plant-Derived Leather

The transition toward circular bio-textiles can be achieved by the use of plant lignocellulosic fibers and pectin-rich residues available from agricultural and food-processing secondary streams.

Unlike synthetic alternatives that rely on petroleum-based polyurethanes, plant-derived substrates utilize the structural integrity of cellulose, lignin, and hemicellulose found in agricultural waste.

Technological advancements in fiber extraction and biopolymeric cross-linking have enabled the development of materials that mimic the grain, breathability, and tensile strength of animal leather. By repurposing high-volume residues, such as pineapple leaf fibers [17,18], apple pomace [19,20], and cactus pads [21], manufacturers have established a circular production model that significantly reduces carbon footprints and landfill loads.

The following commercial examples, that are presented in a text below and in Table 1 represent the vanguard of this transition, successfully translating lab-scale feedstock processing into high-performance, market-ready textiles:

Vegea^®^ is a material characterized by high content of vegetal, renewable and recycled raw materials: grape leftovers from winemaking, vegetal oils and natural fibers from agriculture [22]. The production process utilizes natural biomass, resins, and specifically grape-derived waste to formulate polymeric compounds intended for coated fabrics. These compounds incorporate plant-based additives that give desirable mechanical properties such as elasticity and tensile strength. Grape marc functions not only as a filler but also as an active ingredient due to the presence of tannins and grape seed oil. These ingredients substitute synthetic additives commonly used in coated fabric manufacturing, such as antioxidants, UV stabilizers, chelating agents, and compatibilizers, which are typically derived from non-renewable sources [19].

Vegatex, the US-based company, has several products such as AppleSkin^®^ and LemonSkin™, which are upcycled by-products of apples and lemons from the beverage industry, as well as BarleySkin™, made from brewer’s saved grains [20]. These leather alternatives involve the development of fruit extract-based coated fabrics composed of two polymeric components, prepolymer A and prepolymer B, alongside a textile substrate. Prepolymer A incorporates fruit powder derived from the residual biomass remaining after the extraction of fruit peels and cores, valorizing agricultural waste. It enables partial or complete substitution of conventional polyester components in synthetic leather production, reducing dependence on fossil-derived materials. By integrating fruit-derived residues into the polymer matrix, the process minimizes environmental pollution and contributes to circular resource utilization through the recycling of organic waste streams [23].

Desserto^®^ is a bio-based leather alternative derived from the mature leaves of the *Opuntia* cactus. Its composite structure consists of a compact top layer, a foam intermediate layer, and a textile base, which may be polyester, cotton or a mix of the two. The material is considered sustainable due to the twice-a-year harvesting cycle of the cactus, which requires minimal irrigation and regenerates naturally. The production process involves cleaning and powdering the harvested leaves, followed by sun-drying for approximately three days. Subsequently, proteins and cellulose fibers are extracted and blended to produce the final Desserto^®^ leather material [14,21]

Pinatex^®^, one of the most popular plant-based bio-leathers, is created from the fibers of pineapple leaves, a leftover from the pineapple industry. To generate a non-woven mesh, the fibers from the leaves are removed, cleaned, dried, and combined with polylactic acid (PLA) from maize. This mesh is then coated to create the finished product called Pinatex^®^ [17,21].

**Table 1:** Key attributes of plant-derived leather alternatives.

Name	Feedstock	Processing	Mechanical Properties	Sustainability	biodegradability	Main Applications	References
Vegea^®^	Grape pomace (skins, seeds, stalks)	Drying, mechanical fractionation, oil extraction, polymerization of seed oil, grinding to high fiber powder, then spreading and drying	Tensile strength: 7–10 MPa; Tear strength: 20 N; Elongation: <30%; Flex resistance: 50,000 cycles	Carbon footprint: 4–10 kg CO_2_-eq/m^2^; Toluene detected in hazardous screenings	High biodegradability	Handbags, footwear, fashion accessories, furniture upholstery, automotive interiors	[14,24,25]
Vegatex (AppleSkin^®^)	Apple pomace	Coagulation process or molded pulp technology; mixing pomace with polymers	Tensile strength: 14 MPa; Tear resistance: 18.4 N/mm; Flex resistance: 50,000 cycles	Hazardous screenings detected butanone oxime and traces of DMFa	Not fully biodegradable (contains polyester/PUR blend)	Shoes, luggage, furnishing, upholstery, apparel	[14,25,26]
Desserto^®^	Cactus Opuntia ficus-indica	Reverse coating process; sun-drying pads, grinding, protein extraction, mixing into liquid bio-resin, then pouring on carrier	Tensile strength: 8–25 MPa; Tear resistance: 37.2 N/mm; Elongation: 30–55%; Flex resistance: 30,000 cycles	Carbon footprint: 1.3–2.0 kg CO_2_-eq/m^2^; Hazardous screenings detected butanone oxime, toluene, free isocyanate, folpet, DIBP	Partially biodegradable, varies by backing and coating choice	Handbags, shoes, apparel, furniture, automotive interiors	[14,24,25]
Pinatex^®^	Pineapple leaf fibers	Mechanical extraction of fibers, washing, drying, non-woven mesh formation coated with polymers	Tensile strength: 4.5 MPa; Tear resistance: 31 N/mm; Elongation: 10–20%; Flex resistance: 150,000 cycles	Carbon footprint: 2.7–4.0 kg CO_2_-eq/m^2^; Hazardous screenings detected DIBP	High biodegradability, depending on finish	Footwear, jackets, bags, fashion accessories	[14,24,25,26]

### 4.2. Bacterial Cellulose Leather

**Figure 3:**
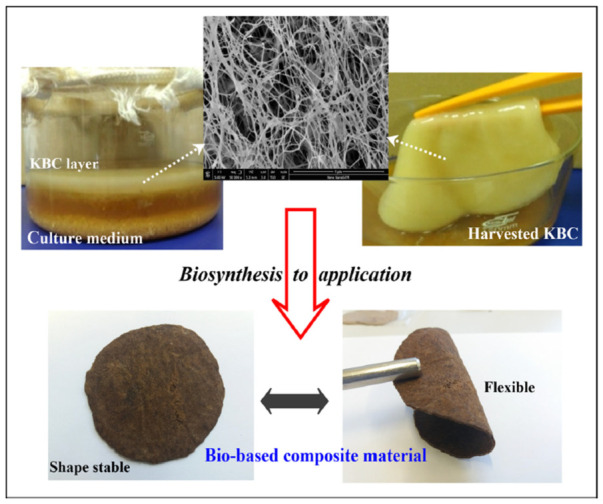
Leather alternative from kombucha-derived bacterial cellulose [32].

Bacterial cellulose (BC) synthesized by microorganisms such as *Gluconacetobacter xylinus*, often via symbiotic cultures of bacteria and yeast (SCOBY), which production ispresented in Figure 3, represents a high-purity, sustainable alternative to traditional animal leather and synthetic leathers. This natural extracellular biopolymer forms a dense, three-dimensional network of interconnected nanometric fibrils characterized by a high crystallinity of approximately 90%, significantly exceeding that of plant-derived cellulose [27,28].

An important advantage of BC is its lack of plant-based impurities such as lignin or hemicellulose as well as a production process that can utilize cost-effective agro-industrial waste and simple fermentation media [28,29,30]. Physical dimensions and mechanical performance of the resulting microbial leather are highly tunable through nutrient selection and post-processing; harvested thicknesses range from 0.129 mm to 0.194 mm, while reconstituted materials have demonstrated the ability to withstand over 100 consecutive folds and achieve tensile strengths of up to 247.21 ± 16.52 N [28,29].

Optimized bio-composite variants, particularly those developed for footwear, exhibit an average tensile strength of 2.13 ± 0.29 MPa and an elastic modulus of 76.93 ± 1.63 MPa, fulfilling the requirements for shoe uppers [31]. Additionally, BC provides functional comfort through improved moisture management, with moisture regain values between 8.7% and 27.0%, ensuring breathability [29]. To address the material’s hydrophilicity—which can result in high swelling ratios—recent research has focused on waterproofing using hydrophobic plant products like *Melaleuca alternifolia* oil and carnauba wax [29,30]. When combined with natural plant-based pigments, these advancements position BC as a durable, biodegradable, and ecologically responsible substrate for high-performance fashion and bio-fabrication [28,29].

### 4.3. Mycelium Leather

**Figure 4:**
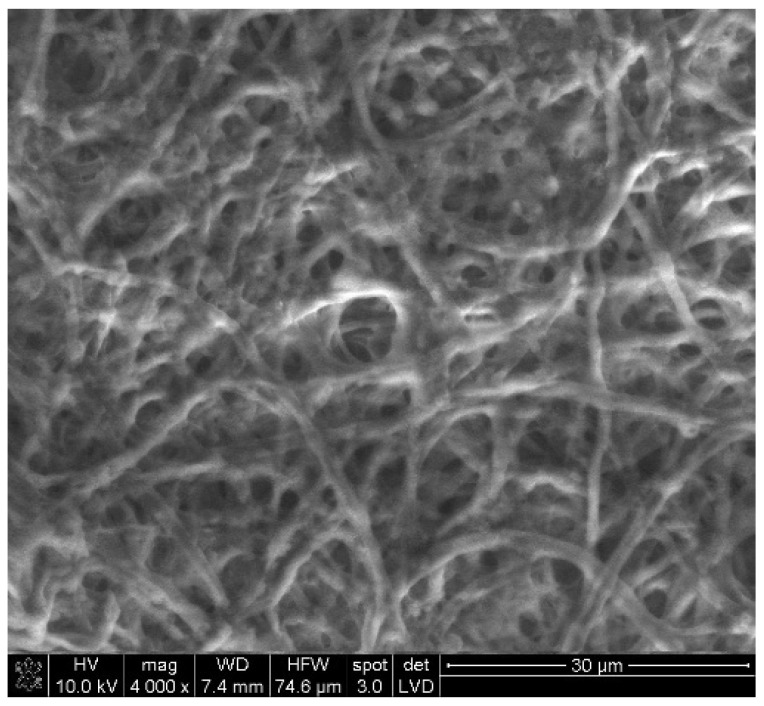
Scanning electron microscope (SEM) image of *Ganoderma lucidum* mycelium.

Ecological materials made of fungi have been gaining increasing interest in recent years. The kingdom of fungi is a biological group with characteristics that set them apart from plants, animals, and bacteria. Fungal cell walls are made of chitin, a material more commonly associated with insect exoskeletons. Their bodies typically consist of hyphae, microscopic filaments that weave together into vast networks called mycelium, (Figure 4), often hidden beneath soil or decaying wood. When the conditions are right, some fungi produce fruiting bodies. Basidiomycete fungi have developed the ability to degrade harsh lignin polymers from wood structures [33].

In recent years, fungal mycelium has gained more attention in fundamental and applied research as well as in innovative commercial platforms, due to its broad application potential, zero waste, and relatively small energy consumption for sterilization during the production process [34].

Fungal characteristics such as growth, cell wall composition and potential to colonize and degrade lignocellulosic feedstock differ among species [35,36] and have a direct influence on properties of the material developed with the use of given fungal species. Other factors that affect fungal material properties are growth substates, growing conditions, such as temperature, humidity and pH [34,36], and the type of fermentation used for cultivation.

Fungal biomass can be cultivated on solid substrates such as agricultural wastes, e.g., straw, sawdust, and rice hulls [37,38,39] and even on natural and synthetic textile waste [40], and the process is called solid-state fermentation. On the other hand, fungi can be cultivated in a bioreactor with the use of liquid-state fermentation [33,41]. In a study carried out by Elsacker et al. [33], liquid-state fermentation was chosen over solid-state fermentation because mycelium mats obtained this way were more homogenous and less prone to primordial formation when grown on a nutritional liquid.

## 5. Most Commonly Used Fungal Species for Leather-like Materials

Thousands of fungal species are distributed globally, but the majority of fungi utilized in the production of mycelium-based materials belong to the phylum *Basidiomycota*. Members of this phylum exhibit efficient colonization and metabolic activity on lignocellulosic substrates, making them particularly suitable for applications involving mycelium composites [36,37].

In Table 2 shown below, we present fungal species that are most often used for mycelium leather production.

**Table 2:** Most used fungal species for mycelium leather-like materials.

Fungal Species/Genus	Common Name	Application Focus	References
*Ganoderma lucidum*	Reishi mushroom	Leather, mats	[33,41,42,43,44,45,46,47,48,49]
*Pleurotus* spp.	Oyster mushrooms	Leather, mats	[38,42,43,44,50,51,52]
*Fomes fomentarius*	Tinder fungus	Leather, mats	[43,50,53]
*Fomitopsis* spp.	Wood decay fungi	Leather, mats	[37,43,50]
*Trametes versicolor*	Turkey tail	Leather, mats	[38,43,45,48,50]
*Agaricus* spp.	Button mushrooms	Leather	[43,50]
*Phellinus* spp.	Wood decay fungi	Leather	[43]

## 6. Production Methods of Mycelium Leather Materials

Production of mycelium-based leather is highly dependent on the selected production methodology, which involves variables ranging from fungal species to environmental cultivation controls. The final material characteristics, such as tensile strength, flexibility, porosity, and aesthetic quality, are influenced by many interdependent factors. These include specific fungal species used, which determine hyphal architecture and growth kinetics; composition and structure of lignocellulosic substrate serving as nutrient base; and cultivation conditions such as temperature, humidity, CO_2_ concentration, and incubation duration. Post-processing treatments, including mechanical pressing, dehydration, chemical crosslinking, and surface finishing, also influence final material properties and quality. Together, these variables define functional and visual properties of the resulting material, positioning mycelium as a versatile substrate for sustainable leather alternatives in fashion, design, and architecture [54]. Production methods, fungal species, and mechanical properties of the obtained materials are summarized in Table 3.

### Examples of Mycelium Leather Production Protocols Developed by Different Scientists

In an article by Crawford et al. [42], mycelium mats were cultivated using *Ganoderma lucidum* and *Pleurotus djamor*, each grown in aluminum trays containing sterilized paste-based media formulated with wheat flour, xanthan gum, malt extract, and additional agents. Grain spawns were evenly distributed on the media under aseptic conditions, and samples were incubated at 22 °C and 40% humidity in darkness for 21 days. Post-harvest treatments included immersion in varying concentrations of glycerol and citric acid, with selected samples undergoing additional tanning using commercial agents. Samples were dried and heat-pressed at 132 °C for 220 s. Selected specimens were dyed with acrylic or spirulina-derived solutions to assess aesthetic potential.

As a result of this study, it was demonstrated that *Ganoderma lucidum* successfully colonized the growth substrate, forming detachable mats with consistent thickness (0.4–0.8 mm, increasing to 1.4–2.58 mm post-treatment), while *Pleurotus djamor* failed to produce viable mycelial sheets due to contamination and inadequate growth. Treatments with higher concentrations of glycerol enhanced flexibility but did not significantly affect tensile strength unless combined with extended tanning, where glycerol concentration showed a statistically significant effect. Citric acid alone did not impact tensile strength. However, subsequent glycerol treatments improved flexibility and microstructure integrity. SEM and visual analyses revealed that prolonged glycerol soaking led to looser fiber networks and better flexibility. Magnesium sulfate contributed to superior material handling and aesthetic qualities by removing glycerol residue and promoting textile-like characteristics, but it did not affect tensile strength.

In an article by Appels et al. [38] *Trametes multicolor* and *Pleurotus ostreatus* were cultivated on sterilized beech sawdust, rapeseed straw, and non-woven cotton substrates under controlled humidity conditions (55–70%) and incubated in autoclavable bags for 14 days. Thermo-formed Polyethylene Terephthalate Glycol (PET-G) molds were then filled with pre-grown substrate, pressed, and covered with perforated foil to facilitate uniform colonization. Growth was extended for an additional 10 days at 25 °C. Final materials underwent heat or cold pressing, followed by ambient or controlled drying. All samples were dried at 80 °C for 24 h and evaluated under standardized ambient conditions.

The results indicated that non-pressed and cold-pressed samples shared similar visual features, with sawdust-based composites appearing denser than those made from straw or cotton. Density measurements ranged from 0.10 to 0.39 g/cm^3^, samples after heat pressing had higher density than those that were not heat-pressed, and their structural variability was also reduced. Heat-pressed mycelium composite materials had better tensile strength and stiffness. Flexural testing confirmed these enhancements across pressing treatments, and cotton-based substrates provided greater elongation at break. Moisture exposure caused weight and dimensional changes primarily during early saturation, with rapeseed-based materials absorbing more moisture than cotton. Water immersion studies showed variable uptake unrelated to fungal strain or processing method, though fungal skin formation influenced hydrophobicity.

Yang et al. [46] used a methodology where *Ganoderma lucidum* was cultivated at 28 °C for 5 days on a substrate blend composed of poplar sawdust, calcium sulfate, water, and wheat flour. Colonized material was fragmented and transferred to a sealed container to promote anaerobic conditions, facilitating further fungal development at 30 °C in darkness for 7 days. The mycelium mat obtained was oven-dried at 60 °C and processed via high-speed mechanical defibrillation to yield a 2% suspension of shredded mycelium fibers (SMFs) and subsequently stored at 4 °C.

**Figure 5:**
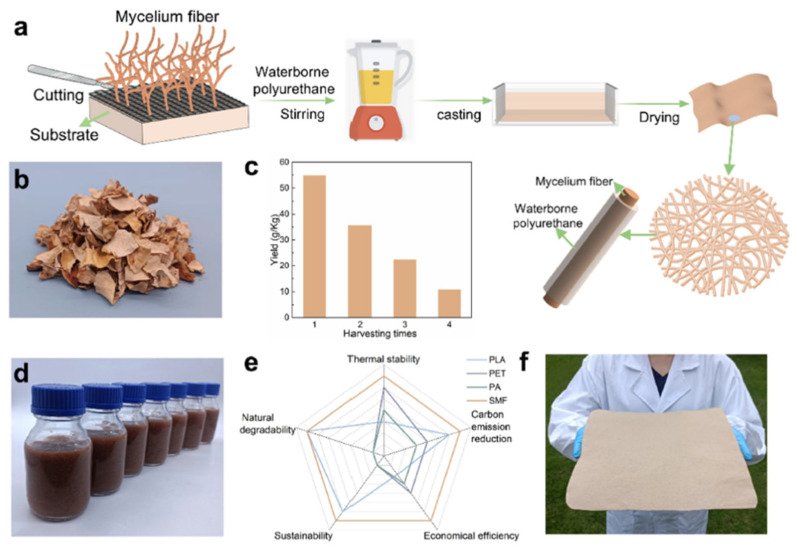
Schematic of fabrication of SMF/WPU leather-like textile. (**a**) Schematic illustration of the fabrication process for branched SMF/WPU leather-like textile using casting and oven-drying methods and the structure of composite textile, in which the SMF was wrapped with WPU. (**b**) Harvested SMF from the poplar wood—*Ganoderma lucidum* inoculum substrate. (**c**) Mycelium yield per batch from 1 kg of dry poplar wood substrate. (**d**) The SMF suspension, defibrillated and dispersed using a kitchen blender. (**e**) Radar chart comparing SMF with commercial PA superfine fibers, PLA superfine fibers and PET superfine fibers. (**f**) Photograph of the large-sized SMF/WPU leather-like textile (28 × 25 × 0.1 cm) [46].

Mycelium composites were prepared by blending shredded mycelium fiber (SMF) suspension with waterborne polyurethane (WPU) at varying weight ratios (10%, 20%, 30%, and 50%), as presented on Figure 5. Each mixture was stirred at 1000 rpm for 5 min at 25 °C and cast into Polytetrafluoroethylene (PTFE) Petri dishes. The suspensions were then oven-dried at 80 °C for 5 h to form uniform films.

The incorporation of shredded mycelium fibers (SMFs) into waterborne polyurethane (WPU) significantly enhanced the mechanical strength of this composite material. Tensile strength increased from 4.59 MPa (WPU) to 18.81 MPa at 50 wt% SMF, while elongation at break decreased from 119% to 5.87%, indicating increased stiffness. The elastic modulus rose with higher SMF content, reflecting improved load-bearing capacity. It was demonstrated that a small strip of SMW-50 (7.4 mm × 1 mm) could support a 1 kg steel block, underscoring the composite’s mechanical strength and potential suitability for sustainable synthetic leather applications.

**Figure 6:**
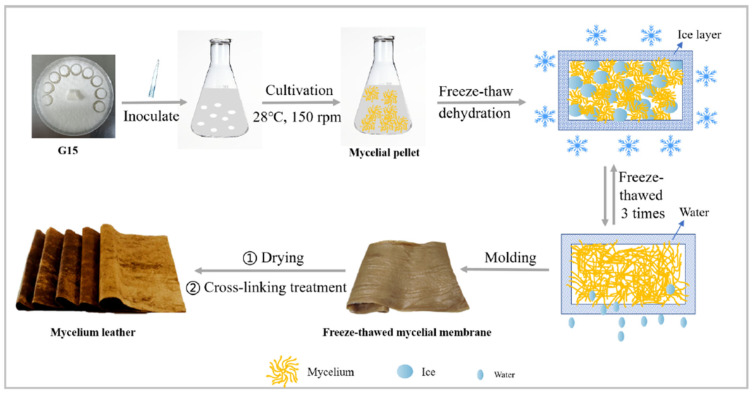
Diagram of mycelium leather preparation [41].

Another approach to the fabrication of mycelium leather material is presented by Song et al. [41] (Figure 6), where *Ganoderma* spp. mycelium was produced through liquid-state fermentation. The liquid fermentation process was chosen to ensure uniform mycelial growth due to precise control of fermentation parameters (pH 5.5, 150 rpm agitation, 28 °C). Post-fermentation, the biomass underwent freeze–thaw dehydration at −7 °C to −80 °C in repeated cycles, followed by mechanical pressing and oven drying to obtain dry mycelium membranes. Selected membranes (−15 FM) were subjected to NaOH alkali treatment for deacetylation, rinsed, and crosslinked with genipin (0.5–1.5%) or tannic acid (3–5%) at elevated temperature. Final treatment included plasticization with a 20% glycerol solution and low-temperature drying to form deep-fermentation mycelium leather mats.

The freeze–thaw method significantly improved dehydration efficiency in fungal membranes, with −15 °C identified as the optimal temperature. At this condition, water content dropped to 47.6% and dehydration peaked at 50.6%, attributed to the formation of evenly distributed, moderate-sized ice crystals that exert mechanical pressure on cell walls, facilitating intracellular water migration and chitin rearrangement. In contrast, temperatures at −40 °C and −80 °C induced rapid freezing, leading to poor ice crystal formation, increased water retention, and reduced porosity. Microscopy revealed that repeated freeze–thaw cycles fragmented mycelial fibers due to structural damage from ice formation.

Crosslinked samples exhibited enhanced softness and elasticity due to the formation of a tightly ordered internal structure. Tensile testing revealed significant increases in both strength and elongation at break, particularly in genipin-treated materials, which achieved maximum values of 6.22 MPa and 18.92%, respectively. This superior performance was attributed to stable imine bond formation and extensive molecular interactions between genipin’s reactive groups and the amino groups in chitosan, resulting in higher crosslinking density compared to tannic acid. Additionally, water contact angle analysis indicated improved hydrophobicity in genipin-crosslinked samples, with smoother, more uniform surfaces likely derived from the controlled formation of ice crystals during processing.

Raman et al. [48], in their study, investigated 14 different fungal species belonging to the order *Polyporales*, called brown rot fungi. Cultures were maintained on YMPA medium at 28 °C. Linear growth was assessed on sawdust–rice bran substrates, while liquid spawn production involved cellophane-covered agar cultures blended and cultured under shaking conditions. Cultivation substrates were transferred into boxes and kept in a high-humidity, CO_2_-enriched environment. Harvested mycelial mats underwent plasticization in glycerol, ethylene glycol and polyethylene glycol and were treated with corn zein and tannic acid. The final step of mycelium mat fabrication was hot-press treatment (60 and 120 °C).

Box cultivation in polypropylene containers under 85% humidity and elevated CO_2_ (1500 ppm) revealed significant mat variation across strains: while *E. applanata* and *T. hirsuta* failed to produce viable mats, *F. fraxinea* yielded cohesive, brownish-white, high-density mycelium with 12.08% ± 2.91% substrate-to-product yield efficiency.

Mechanical analysis revealed that 20%-PEG-treated mats displayed superior tensile strength and elasticity, with Young’s modulus exceeding 8 MPa in hot-press-treated samples. SEM imaging showed that treated mats developed uniform hyphal architectures and compact cell wall arrangements. The results indicated *F. fraxinea* as a promising fungal candidate for mycelium leather applications, demonstrating optimal growth, mat cohesion, and favorable mechanical properties.

**Table 3:** Comparison of mycelium materials from chosen research articles.

Feature	Mycofabrication of Mycelium-Based Leather from Brown-Rot Fungi (Raman et al., 2022) [48]	Revolutionizing Eco-Friendly Leather Production: A Freeze-Thaw and Liquid Fermentation Approach with Fungal Mycelium (Song et al., 2025) [41]	Fabrication Factors Influencing Mechanical, Moisture- and Water-Related Properties of Mycelium-Based Composites (Appels et al., 2019) [38]	Growing Mycelium Leather: A Paste Substrate Approach with Post-Treatments (Crawford et al., 2024) [42]	Scalable Production of Robust, Moisture-Wicking, and Breathable Superfine Mycelium Fiber/Waterborne Polyurethane Leather-like Textile via Direct Casting and Oven-Drying (Hao et al., 2025) [46]
Fungal Species	Multiple Polyporales (e.g., *Ganoderma lucidum*, *Fomitella fraxinea*)	*Ganoderma lucidum*	*Trametes multicolor*, *Pleurotus ostreatus*	*Ganoderma lucidum*, *Pleurotus djamor*	*Ganoderma lucidum*
Substrate	Oak sawdust and rice bran	Liquid culture with glucose and yeast extract	Rapeseed straw, beech sawdust and cotton	Paste substrate (flour, malt extract, xanthan gum, cream of tartar, citric acid)	Poplar sawdust, CaSO_4_, flour
Cultivation Method	Solid-state fermentation in boxes	Liquid fermentation in bioreactor	Solid-state on pressed molds	Paste-based solid-state fermentation in trays	Substrate-based growth in vented polyethylene bags, incubated and peeled, then defibrillated
Processing	PEG plasticization, coating, cross linking and hotpressing	Freeze–thaw cycles at −7, −15, −40, or −80 °C, deacetylation, crosslinking, plasticization	No pressing, cold pressing, or heat pressing	plasticization, crosslinking, tanning, hot pressing, dyeing	mycelium blending, casting, oven-drying
Chemical Agents Used	PEG (20%), glycerol, ethylene glycol, corn zein, tannic acid	NaOH for deacetylation, genipin and tannic acid (3–5%) for crosslinking, glycerol (20%) for plasticization	None specified for plasticizing; physical pressing only	Glycerol, citric acid, magnesium sulfate, commercial tanner, heat pressing	Mixed with waterborne polyurethane (WPU), oven-dried
Mechanical Properties	Tensile Strength: 8.49 ± 0.9 MPa Elastic Modulus: 8.14 MPa ± 0.88 MPa	Tensile Strength: 6.22 MPa Elongation at Break: 18.92%	Tensile Strength: 0.24 ± 0.03 MPa Elastic Modulus: 97 ± 9.0 MPa	Tensile Strength: Max ~1.4 N/cm^2^ (0.14 MPa)	Tensile Strength: 18.81 MPa Elastic Modulus: 1.81 MPa
Density	1.35–1.46 g/cm^3^	Not specified	0.10–0.39 g/cm^3^	Not specified	Not specified

## 7. Comparison of Physical Properties of Mycelium Materials with Bovine Leather, Synthetic PU Leather and Commercially Available Bio-Leather Alternatives

Table 4 presents a comparative analysis of physical and mechanical properties of leather, PU-coated textile and commercially available bio-based leather alternatives. Commercial materials such as vegetable-tanned bovine leather, PU-coated textiles, Appleskin^®^, and SnapPap^®^ exhibit high tensile strength (up to 32.32 N/mm^2^) and tear resistance, making them suitable for applications in fashion, upholstery, and accessories. Breathability and water vapor absorption vary widely, with Muskin^®^ and SnapPap^®^ demonstrating superior moisture management.

Mycelium-based leathers show promising but variable performance. Most fungal composites remain below 1 N/mm^2^ in tensile strength, except for Mylea^TM^ and mycelium and waterborne polyurethane (WPU) composites, where tensile strength was up to 11 N/mm^2^ and 18.81 N/mm^2^, respectively. Processing methods, including freeze–thaw cycles, Polyethylene Glycol (PEG) plasticization, and hot pressing, significantly influence material performance.

While commercially available bio-leathers offer consistent performance and scalability, some mycelium-based materials are approaching industrial viability. Hybridization with bio-based polymers and scalable post-treatment techniques may bridge the gap between laboratory innovation and market readiness, positioning fungal composites as sustainable contenders in the future of eco-leather production.

**Table 4:** Comparison of physical properties of leather and commercially available leather alternatives.

Physical Properties	Thickness [mm]	Tensile Strength [N/mm^2^]	Tear Resistance [N/mm]	Water Vapor Permeability [mg/(cm^2^ h)]	Water Vapor Absorption [mg/cm^2^]	References
Bovine Leather chromium-tanned	1.27	25.39	41.11	4		[55]
Bovine leather vegetable-tanned	2.2	32.32	67.5	1.1		[55]
Muskin^®^	6.22	0.2	0.5	10.4	6	[14]
Kombucha	0.29	9.7	5.1	0.1	9.2	[14]
PU coated textile	1.11	10.2	17	1.1	1.4	[14,56]
Desserto^®^	1.28	9.48	47.74			[55]
Appleskin^®^	1.14	14	18.8	0.4	1.7	[14]
Vegea^®^	0.95	9.4	16.6	0.6	3	[14]
Teak Leaf^®^	0.57	12.2	30.7	0.1	0.1	[14]
Pinatex^®^	1.59	5.1	68.12	6.8		[55]
SnapPap^®^	0.57	24.9	7.5	10.3	3.7	[14]
Leatherette	0.34	5.5	7.12	0.4		[55]
Mylea^TM^	3–9	8–11	24	n/a	n/a	[57]

## 8. Applications of Mycelium Materials Across Industries

Materials derived from fungal biomass are gaining attention as a sustainable alternative to animal and synthetic leather with applications that extend beyond the fashion industry. Their versatility and eco-friendly properties enable a wide range of potential applications, such as automotive interiors, packaging, construction, electronics, and biomedical engineering.

The most important application areas include:

Fashion and textiles: Mycelium leather is used in clothing, footwear, bags, and accessories, offering a biodegradable and cruelty-free alternative to traditional leather. Popular fashion brands (e.g., Hermès, Stella McCartney, Adidas) are prototyping products using mycelium leather produced by companies such as Bolt Threads and Mycoworks [44,49,52,55,56,58].

Automotive and interior design: Mycelium leather is explored for car seat covers, upholstery, and interior panels, providing a sustainable option for the automotive industry [44,55]. Mycelium composite’s customizable form, texture and durability are very good for interior design applications [52,59,60,61].

Packaging and construction: Mycelium materials are used as eco-friendly packaging, replacing expanded polystyrene and other plastics. In the construction industry, mycelium composites serve as insulation, sound-absorbing panels, and lightweight building materials due to their fire resistance and thermal properties [52,58].

Electronics: Mycelium materials are being tested as substrates for electronic circuit boards, e-textiles, and reactive wearables [55,58].

Medicine and cosmetology: Mycelium-based foams and films are being investigated as sustainable, skin-compatible materials for use in cosmetic pads, facial masks, and applicators due to their effective moisture regulation and hypoallergenic properties. Their porous structure offers potential for the controlled release of active cosmetic ingredients, although this remains a conceptual application under early-stage exploration [58,62,63,64].

## 9. Challenges

Bio-based leather materials are a very promising sustainable alternative to animal leather and to synthetic materials; however, there are some challenges that need to be addressed. The main challenges are:

Mechanical properties of mycelium leather are still inferior in comparison with animal leather. Untreated mycelium is brittle and requires post-treatment such as cross-linking and plasticizing to improve material performance. Additionally, quite often, mycelium leather is made by mixing mycelium with petroleum-based non-biodegradable compounds in order to achieve better mechanical properties. Such materials often do not meet the biodegradability criteria [48,49,50,59]. Materials derived from plant waste show moderate strength and limited flexibility compared to bovine and PU leathers. For example, pineapple-leaf fiber alternatives can be stiff with limited drape, while grape-based leathers exhibit intermediate mechanical performance—stronger than unreinforced cellulose but less robust than reinforced versions [24,26].

Water adsorption can cause irreversible changes in mycelium material properties, influencing its durability and usability. This is mostly because additional polymeric components of mycelium leather are sensitive to moisture, which can reduce final products’ elasticity and mechanical strength [65,66]. Plant fibers and microbial cellulose are hydrophilic, thus sensitive to water adsorption, swelling, and dimensional instability [24,67]. While applying hydrophobic surface agents, e.g., waxes or oils, can improve water resistance, these treatments can sometimes be compromised by repeated flexing or may inhibit the material’s water vapor permeability (breathability), reducing consumer comfort [14,24,28].

The scalability of bio-based leather materials has various technical and economic challenges. The main obstacle is achieving consistent material quality, as mycelium growth greatly depends on environmental variables such as temperature, humidity, and substrate composition [42,44,46,68], whereas plant-based materials, such as grape pomace, are often seasonal and geographically limited to specific regions [24]. This can lead to differences in final material texture, thickness, and mechanical properties as well as create supply chain instability and cost variability.

The high production cost of mycelium and bacterial leather material is primarily due to the complexity of cultivation and processing which requires specialized and expensive infrastructure. Mycelium must be grown under sterile, highly controlled conditions; it requires precise temperature and humidity regulation, as well as substrate composition. Microbial cellulose requires specific fermentation conditions and long processing times, which can increase infrastructure and labor costs [27,30]. Post-processing steps such as plasticizing, crosslinking and hot-pressing increase labor and energy costs [69,70,71].

Life Cycle Assessments (LCAs) of plant-based and microbial leather alternatives have shown their significant ecological advantages over synthetic petroleum-based materials like polyurethane (PU), although their impact depends on production methods and energy sources. Plant-derived materials, such as cactus leather, have high sustainability with a carbon footprint as low as 1.4 kg CO_2_ eq/m^2^ and minimal water consumption (0.02 m^3^/m^2^), though the common use of PU coatings for durability remains an obstacle for full biodegradability. Similarly, bacterial cellulose (SCOBY) and mycelium offer big circular economy potential by utilizing agricultural waste as substrates. However, mycelium-based materials exhibit a wide range of environmental profiles: while energy-intensive processes relying on fossil fuels can result in a footprint of 57.15 kg CO_2_ eq/m^2^ (e.g., Mylea™), optimized passive cultivation using renewable energy and biodegradable adhesives can reduce this to just 2.76 kg CO_2_ eq/m^2^ (e.g., Reishi™). Ultimately, while these alternatives can be up to 1000% more sustainable than animal leather, their true environmental success depends on the transition to clean energy, the elimination of synthetic glue, and the avoidance of plastic-based finishing agents [72,73,74].

Material biosafety is an important aspect that should be considered during the development of mycelium leather. Since fungi are living organisms, certain species can be pathogenic and cause diseases. Additionally, some fungi may produce mycotoxins—harmful secondary metabolites that pose risks through ingestion, inhalation, or dermal contact. Another concern is the potential release of fungal spores, which can trigger allergic reactions when inhaled. However, the fungi that are most commonly used in mycelium leather production, such as *Ganoderma*, *Pleurotus*, and *Trametes*, are generally regarded as safe and non-pathogenic. The manufacturing process, particularly heat treatment and drying, inactivates fungal growth and minimizes spore release, significantly reducing biological hazards.

When appropriate fungal strains are selected and production conditions are well-controlled, mycelium materials present a low biosafety risk. Nevertheless, standardized testing protocols and further research are essential to confirm their safety across long-term exposure and large-scale industrial applications [73,75].

Additionally, there is a need for future research that will confirm the biocompatibility of microbial cellulose and natural dyes to ensure they do not cause allergenic or toxic effects when in long-term contact with human skin [29].

## 10. Conclusions

Bio-based materials, including plant-derived and microbial leather alternatives, play an important role in the search for sustainable replacements for bovine leather and petroleum-based synthetics like PVC and PU. Plant-based alternatives utilize diverse agricultural by-products, including pineapple leaf fibers, cacti, grape pomace, and apple waste, to create durable composites that effectively upcycle agro-industrial waste. Similarly, microbial materials, such as fungal mycelium and bacterial cellulose, offer renewable and biodegradable options that can be grown into specific shapes, significantly reducing material waste during the manufacturing of fashion and medical goods. Both categories offer substantial ecological advantages, such as a lower carbon footprint and the elimination of toxic chromium-tanning processes.

However, despite these advantages, both material types face significant technical and regulatory problems. The main challenges remain mechanical strength, water resistance, and long-term durability, particularly in load-bearing or outdoor applications. While some plant-based fibers, such as pineapple leaf fibers, exhibit high tensile strength, many commercial versions still rely on petroleum-derived resins or coatings to achieve the necessary durability, which can compromise their total biodegradability. For microbial and mycelium leathers, ensuring biological safety is most important; rigorous post-processing is required to reduce risks such as spore release or mycotoxin contamination. Furthermore, for all bio-based materials, there is a need for standardization in production processes, physical testing, and international regulatory frameworks to ensure material consistency.

In summary, both plant-based and microbial materials have great potential as environmentally friendly biomaterials, but existing technical, economic, and perception-related challenges should be addressed. Future research and development should prioritize the elimination of synthetic binders and the enhancement of natural cross-linking methods to fully align with a circular economy. Continued innovation in these fields is very important to provide good quality, ethically responsible materials that meet the growing global demand for sustainable fashion and design.

## Data Availability

No new data were created or analyzed in this study. Data sharing is not applicable to this article.

## Abbreviations

The following abbreviations are used in this manuscript:

PEG	Polyethylene Glycol
WPU	Waterborne Polyurethane
SMFs	Shredded Mycelium Fibers
PET-G	Polyethylene Terephthalate Glycol
BOD	Biochemical Oxygen Demand
PVC	Polyvinyl Chloride
PU	Polyurethane
COD	Chemical Oxygen Demand
SCOBY	Symbiotic Cultures of Bacteria and Yeast
BC	Bacterial Cellulose
SEM	Scanning Electron Microscope
LCA	Life Cycle Assessment
PLA	Polylactic Acid
